# Constitutional and somatic deletions of the Williams-Beuren syndrome critical region in Non-Hodgkin Lymphoma

**DOI:** 10.1186/s13045-014-0082-4

**Published:** 2014-11-07

**Authors:** David Guenat, Samuel Quentin, Carmelo Rizzari, Catarina Lundin, Tiziana Coliva, Patrick Edery, Helen Fryssira, Laurent Bermont, Christophe Ferrand, Jean Soulier, Christophe Borg, Pierre-Simon Rohrlich

**Affiliations:** Laboratory of Cellular and Molecular Biology, University Hospital of Besançon, 1 Bd Fleming, Batiment Diaclone, Besançon, France; UMR1098 Inserm/EFS-BFC/University of Franche-Comte, LabEx LipSTIC, Besançon, France; Saint-Louis Hospital APHP and Hematology University Institute (IUH), University Paris-Diderot, Paris, France; Department of Pediatrics, San Gerardo Hospital, University of Milano-Bicocca, Monza, Italy; Department of Clinical Genetics, University and Regional Laboratories Region Skane, Lund University, Lund, Sweden; Department of Genetics, Hospices Civils de Lyon, Bron, France; Department of Medical Genetics, Medical School, University of Athens, Athens, Greece; Biochemistry Laboratory, University hospital of Besançon, Besançon, France; Department of Medical Oncology and CIC-BT506, University Hospital of Besançon, Besançon, France; Department of Pediatric Hemato-Oncology, University Hospital of Nice, Nice, France

**Keywords:** Williams-Beuren syndrome, Non-Hodgkin Lymphoma, 7q11.23, Cancer predisposition, DNA repair

## Abstract

Here, we report and investigate the genomic alterations of two novel cases of Non-Hodgkin Lymphoma (NHL) in children with Williams-Beuren syndrome (WBS), a multisystem disorder caused by 7q11.23 hemizygous deletion. Additionally, we report the case of a child with NHL and a somatic 7q11.23 deletion. Although the WBS critical region has not yet been identified as a susceptibility locus in NHL, it harbors a number of genes involved in DNA repair. The high proportion of pediatric NHL reported in WBS is intriguing. Therefore, the role of haploinsufficiency of genes located at 7q11.23 in lymphomagenesis deserves to be investigated.

**Electronic supplementary material:**

The online version of this article (doi:10.1186/s13045-014-0082-4) contains supplementary material, which is available to authorized users.

## Findings

Chromosomal disorders are common circumstances for the discovery of a genetic predisposition to cancer allowing identification and localization of new oncogenes. Williams-Beuren syndrome (WBS) is a multisystem disorder caused by 7q11.23 hemizygous microdeletion [1]. WBS is not currently considered as a risk factor for cancer. However, the low incidence of both WBS and pediatric NHL (Non-Hodgkin Lymphoma) might hamper our ability to identify any association between these rare diseases. An increased risk of NHL in WBS might have easily been underestimated until today. Furthermore, the number of pediatric cancer reported in WBS has reached 11 cases and, strikingly, 8 (73%) of them where NHL, mostly Burkitt lymphoma (Table 1). Here, we report 2 novel cases of NHL in children with WBS and the additional case of a non-WBS child with NHL and somatic 7q11.23 deletion (see case description in Additional file 1).

**Table 1:** **List of pediatric cancers reported in WBS patients**

	**Study Samples**	**Authors**	**Date of publication**	**Age (years), gender**	**Type of tumor**	**Reference**
1	WBS Patient 1	Guenat D	TS	7, F	NHL (Burkitt)	-
2	WBS Patient 2	Guenat D	TS	10, M	NHL (B-NHL stage IV)	-
3	-	Vanhapiha N	2014	7, M	NHL (Burkitt) and Ewing sarcoma	[2]
4	-	Chonan M	2013	3, M	Astrocytoma	[3]
5	-	Zhukova N	2013	8, M	NHL (Burkitt)	[4]
6	-	Onimoe G	2011	10, F	NHL (Burkitt)	[5]
7	-	Urisarri Ruiz A	2008	12, M	NHL (T-cell)	[6]
8	-	Thornburg CD	2005	1,?	NHL (Burkitt)	[7]
9	-	Amenta S	2004	8, M	NHL (Burkitt)	[8]
10	-	Culic V	2002	14, M	ALL	[9]
11	-	Semmekrot BA	1985	5, ?	Astrocytoma	[10]

TS: This Study; NHL: Non-Hodgkin Lymphoma; ALL: Acute Lymphoblastic Leukemia, F: female; M: male.

**Figure 1:**
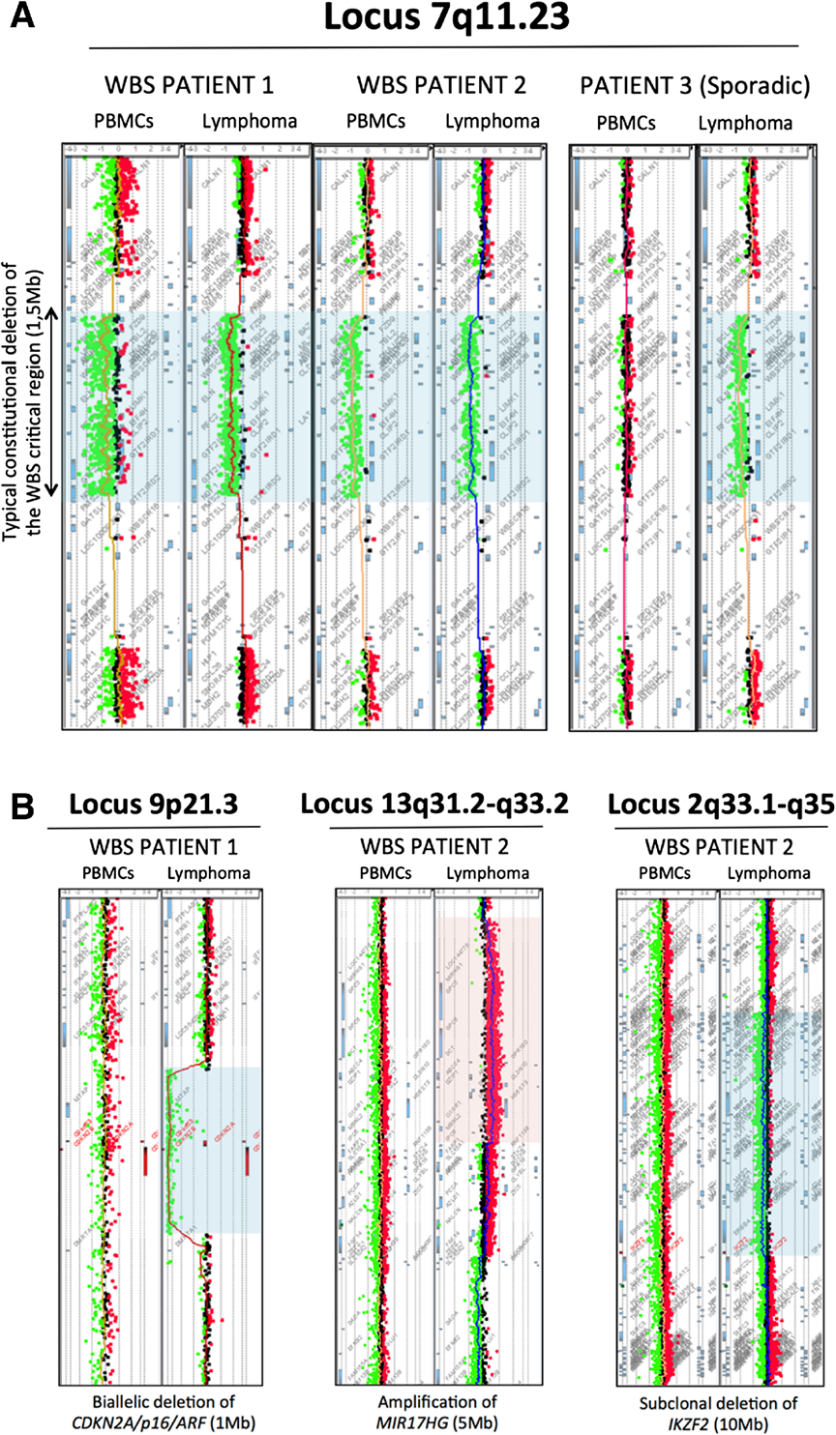
**Array-based comparative genomic hybridization analysis of normal tissues and lymphoma cells of 2 WBS patients and of a third child without WBS but with NHL and a somatic 7q11.23 deletion. A**. Typical hemizygous loss of the WBS region on chromosome 7q11.23 spanning 1.5 Mb was observed in normal and tumor DNA of 2 patients with WBS. Patient 3 had the same deletion in lymphoma cells but this deletion was somatic. **B**. Study of somatic chromosomal rearrangements showed a homozygous deletion encompassing chromosome band 9p21.3 at the INK4a/ARF locus on the tumor of patient 1. WBS patient 2 exhibited a large subclonal deletion on chromosomal region 2q33.2-q34 encompassing the *IKZF2* gene locus and an amplification of a 10 Mb region at the locus 13q31.2-q31.3 involving *MIR17HG*. Patient 3 had no other chromosomal rearrangements in the tumor. PBMC: Peripheral Blood Mononuclear Cells.

Since several different deletions account for the genotype of WBS, we first investigated germline and somatic structural variants in these latest 3 patients using array-based CGH (Figure 1). We confirmed the presence of a constitutional 7q11.23 deletion in WBS patients. No germline Copy Number Variation (CNV) was observed in patient 3. Intriguingly, the somatically acquired 7q11.23 deletion that occurred in lymphoma of patient 3 was similar to the classical germline deletion observed in WBS patients and there was no evidence of other rearrangements occurred in this tumor. Lymphoma cells of WBS patients exhibited CNV commonly found in B cell lymphoma excepting a subclonal deletion of approximately 10 Mb at the locus 2q33.1-q35 involving *IKZF2* in WBS patient 2. A recent study by Holmfeldt et al. demonstrated that this deletion was common in low-hypodiploid acute lymphoblastic leukemia [11] but this deletion had never been described in NHL. Then, next generation sequencing of the WBS region showed a germline homozygous variant in exon 25 of *ELN* (c.1741G > C, p.Gly581Arg, rs.17855988) in the 2 WBS patients. *ELN* encodes elastin, a constituent of elastic fibers. Many variants of *ELN* has been described and were associated to the severity of cardiovascular symptoms of WBS patients. No recurrent variant was observed in the 27 other genes nor in the 2 miRNA loci of the WBS critical region.

A number of genes mapping to the WBS region are involved in DNA repair: 1) Eleven *PMS2* pseudogenes loci are located at 7q11.23. PMS2 plays a crucial role in the DNA Mismatch Repair and a childhood cancer syndrome is associated to biallelic mutations of *PMS2* [12]. However, in our study the 3 patients showed stable microsatellites (Additional file 2); 2) *BAZ1B* encodes a transcription factor with an intrinsic tyrosine kinase domain that phosphorylates Tyr142 of histone H2A.X and is involved in the maintaining of gH2A.X in the sites of DNA damages [13]; 3) *RFC2* encodes a subunit of the Replication Factor C complex that interacts with BRCA1 for post replication repair after UV-induced DNA damage [14]; 4) *GTF2I* encodes a transcription factor that promotes DNA translesion synthesis and genomic stability interacting with PCNA and DNA polymerases [15]. Finally, the hypothesis of a constitutional genomic instability in WBS is consistent with the results of a study by Savina et al. that showed experimentally the relationship between an abnormal DNA-damage response and the 7q11.23 hemizygous microdeletion when comparing the comet assay data in FISH-positive and FISH-negative lymphocytes from WBS-suspected patients [16].

7q11.23 deletion has been found as a relatively common occurrence in pilocytic astrocytoma [17], of which variants have been reported in 2 patients with WBS [3,10]. Nevertheless, genome-wide copy number analysis in NHL, including a recent study by Conde et al. that analyzed 648 patients, ages 20 to 85 years, have not found a susceptibility locus at 7q11.23 [18]. However, NHL in adults encompasses a heterogeneous spectrum of diseases in which diffuse large B-cell lymphoma predominates. NHL in children is a more rare event and pediatric DLBCL is uncommon. Burkitt lymphoma that predominates in pediatric NHL, has also been studied by genome-wide CNV studies. Notably, Scholtysik et al. demonstrated a recurrent loss at 7q11.22, localized at the centromeric limit of the WBS critical region, in 39 cases of BL [19]. Amplifications of the WBS critical region have also been found in a variety of cancers including large B-cell lymphoma [20], ovarian adenocarcinoma [21], papillary thyroid carcinoma [22] and cholangiocarcinoma [23]. This might reflect complex mechanisms that regulate initiation/promotion of cancer cells by oncogenes and tumor suppressor genes clustered around recombination hot spots or fragile sites in the WBS region.

Althought no epidemiological studies demonstrated an increased risk of cancer in WBS the high proportion of pediatric NHL reported in WBS and the occurrence of a somatic deletion of 7q11.23 in NHL is intriguing. In these patients, NHL seems to arise in the presence of the typical WBS microdeletion and in the absence of homozygous mutation. The role of haploinsufficiency of genes located at 7q11.23 in lymphomagenesis deserves to be investigated.

## Additional files

Additional file 1: **Supplementary materials and methods [** 24 **].**

Additional file 2: **Study of microsatellites instability by capillary electrophoresis fragment analysis.** All patients have stables microsatellites in both normal and tumor DNA when compared with the Microsatellite Instable positive control (Control MSI+). BL: Burkitt Lymphoma, PBMC: Peripheral Blood Mononuclear Cells.
