# Supplementary material for: Constitutional and somatic deletions of the Williams-Beuren syndrome critical region in Non-Hodgkin Lymphoma
Source: J Hematol Oncol. 2014 Nov 7;7:82. doi: 10.1186/s13045-014-0082-4 (PMC4228180; doi:10.1186/s13045-014-0082-4)
Supplement: Supplementary file 1 — Supplementary materials and methods [ 24 ]. [file 13045_2014_82_MOESM1_ESM.doc]

**SUPPLEMENTARY MATERIAL AND METHODS**

**Patients**

WBS Patient 1 is a girl with typical WBS features confirmed by Fluorescence In Situ Hybridization. At the age of seven, she developed asthenia, weight loss and an abdominal mass. CT scan revealed mesenteric, periaortic and coeliac lymphadenopathies, hepato-splenomegaly and ascites. Lactate dehydrogenase (LDH) level was markedly increased (5059 U/ml). Cytological, immunophenotypic and cytogenetic studies of the ascites fluid confirmed the diagnosis of Burkitt Lymphoma with 46,XX,t(8;14)(q24;q32), composed of a monotypic lymphoid cell population (CD19+, CD20+, CD10+, CD22+, FMC7+, CD5-, CD23-, sIg+ kappa light chain restriction). A polychemotherapy treatment according to the LMB 2001 protocol was undertaken. At the time of this report, patient 1 has been off therapy for 5 years and remains in complete remission.

WBS patient 2 is a boy with WBS who presented at the age of ten with a two-week history of fever and cervical lymphadenopathy. Biochemistry showed a high LDH value (1059 U/ml). Progressive joint pain, walking impairment and neurogenic bladder were also noted. The patient underwent a bone marrow aspiration and was diagnosed with a B-cell mature acute lymphoblastic leukemia with translocation t(8;14)(q24;q32). A magnetic resonance imaging (MRI) of the spine showed an epidural pathologic tissue between lumbar vertebra 3 and 4, suggesting leukemic infiltration. The patient was treated according to the intensive chemotherapy protocol of the Italian Association of Pediatric Hematology and Oncology (AIEOP) NHL 97 protocol. He achieved a complete morphological remission after the first chemotherapy cycle. At the time of this report, the patient is 14 years old and he remains in complete remission.

Patient 3 is a 12-year-old child diagnosed with BL without WBS clinical features. Lymphoma cells from this patient have shown a 7q11.23 deletion in a previous study of cytogenetic abnormalities in sporadic BLs.

**Samples**

Peripheral blood mononuclear cells (PBMC) or frozen bone marrow mononuclear cells isolated after remission of the 3 patients were used to extract genomic DNA. Tumor-derived DNA was isolated from ascites fluid for patient 1, frozen bone marrow mononuclear cells for patient 2 and from the abdominal mass for patient 3. Tumor cell content was 80%, 60% and 52% for patients 1 to 3 respectively. DNA was isolated from tissues using QIAamp DNA Mini Kit (Qiagen) according to the manufacturer’s instructions.

**Array-based Comparative Genomic Hybridization**

Oligonucleotide array-CGH was performed using the SurePrint G3 Human CGH Microarray Kit, 4x180K (Agilent Technologies). In the 180K kit a total of 180 880 probes are included with an overall median probe spacing of 13 Kb. Experiments were performed according to the standard Agilent protocol. Commercially available genomic DNA (Promega) was used as a control. Hybridized slides were scanned with a microarray scanner (G2505B SureScan High-Resolution Technology Agilent), and the image data were extracted and converted to text ﬁles using Agilent Feature Extraction software. The data were graphed and analyzed using Agilent CGH Analytics software (statistical algorithm: ADM-2; sensitivity threshold: 6.1).

**Next Generation Sequencing**

Targeted sequencing was performed using Ion Torrent PGM (Life Technologies). A total of 728 primer pairs targeting the CDS and UTR of 27 genes (*ABHD11, BCL7B, BAZ1B, CLDN3, CLDN4, CLIP2, DNAJC30, EIF4H, ELN, FKBP6, FZD9, GTF2I, GTF2IRD1, GTF2IRD2, LAT2, LIMK1, MLXIPL, NCF1, NSUN5, RFC2, STX1A, TBL2, TRIM50, VPS37D, WBSCR22, WBSCR27, WBSCR28*) and 2 miRNA loci (has-mir-590 and has-mir-4284) mapping on critical region of WBS were designed online with ampliseq designer (www.ampliseq.com).

Libraries prepared with Ion torrent AmpliSeq 2.0 Beta Kit (Life Technologies) for each sample with distinct barcoding were diluted and pooled and were subjected to emulsion PCR using IonTorrent One Touch template kit accordingly with the manufacturer’s instructions. A Quality control was performed using Qubit Ion sphere kit to estimate the percentage of Ionsphere with DNA. Ionspheres were then enriched with IonTorrent OneTouch kit and loaded on an Ion 316 chip to sequence pooled libraries of 4 samples. A cut-off point of 300,000 reads with a quality score of AQ20 was used as a measure of successful sequencing. A sequence variant was considered as authentic if the coverage was superior to 1000X. The sequences were aligned to human genome build 19 reference genome and identification of variants was achieved using IonTorrent VariantCaller plugin software version 1.0. The Integrative Genome Viewer (IGV) was used to visualize the reads alignments and the presence of variants against the reference genome.

**Microsatellite instability analysis**

Microsatellite instability was investigated using a PCR-based method to analyze the consensus genomic markers BAT25, BAT26, NR21, NR24 and NR27. Primer sequences described by Buhard et al. were used[24]. The five markers were coamplified in a multiplex PCR using the 2x Multiplex PCR Master Mix (Qiagen) (denaturation at 95°C for 15 minutes, 30 cycles of denaturation at 94°C for 30 seconds, annealing at 62°C for 90 seconds, and extension at 72°C for 60 seconds, followed by an extension at 72°C for 10 minutes) and were analyzed on an CEQ 8000 GexP Genetic analysis system (Beckmann Coulter) according to manufacturer’s instructions.
