# Supplementary figures and images for: Constitutional and somatic deletions of the Williams-Beuren syndrome critical region in Non-Hodgkin Lymphoma
Source: J Hematol Oncol. 2014 Nov 7;7:82. doi: 10.1186/s13045-014-0082-4 (PMC4228180; doi:10.1186/s13045-014-0082-4)

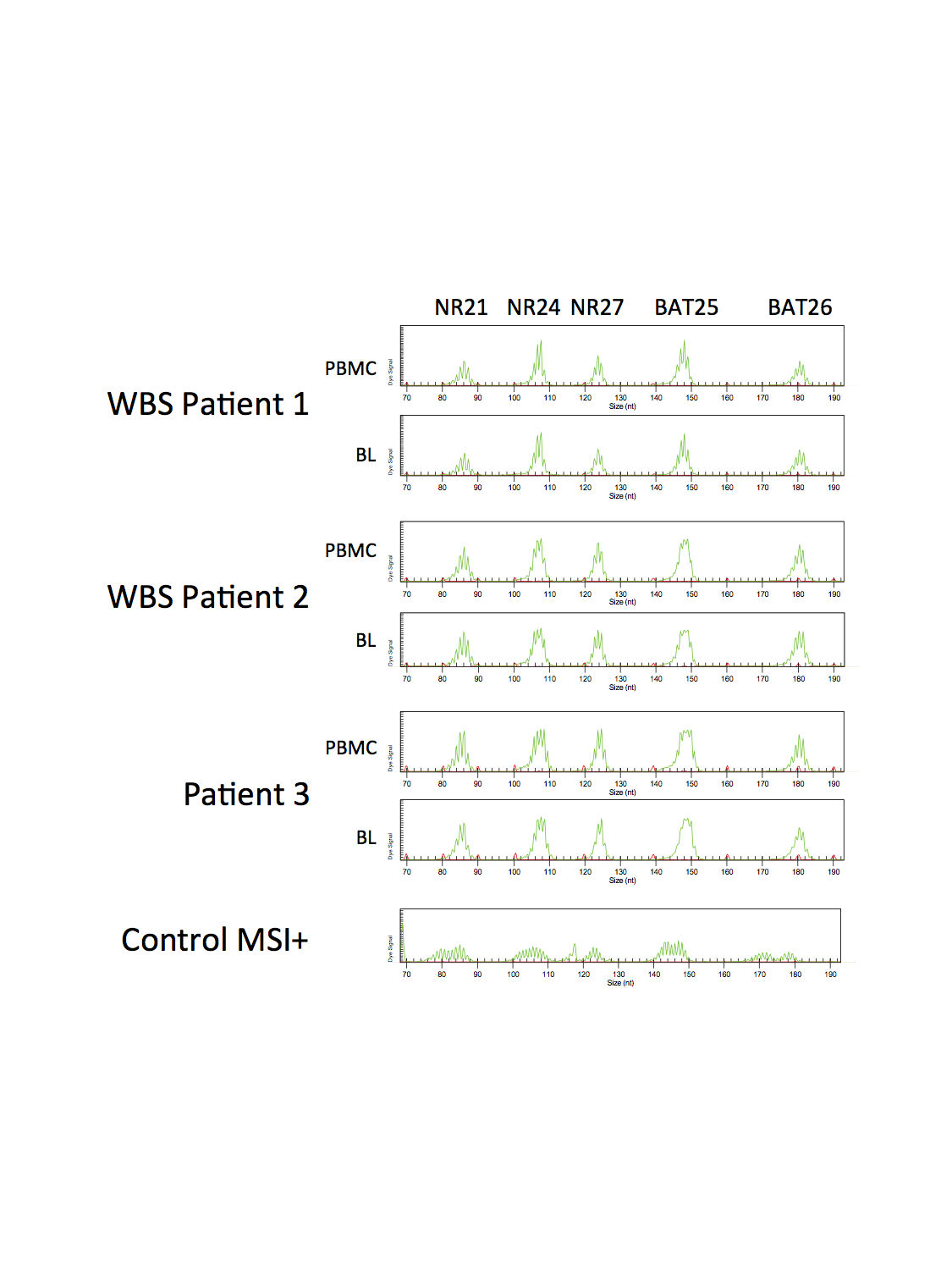

Supplement: Supplementary file 2 — Study of microsatellites instability by capillary electrophoresis fragment analysis. All patients have stables microsatellites in both normal and tumor DNA when compared with the Microsatellite Instable positive control (Control MSI+). BL: Burkitt Lymphoma, PBMC: Peripheral Blood Mononuclear Cells. [file 13045_2014_82_MOESM2_ESM.png]
